# Treatment Persistence and Factors Associated with Bimekizumab Discontinuation in Psoriatic Arthritis and Axial Spondyloarthritis: A Real-World Multicenter Study

**DOI:** 10.3390/biomedicines14081778

**Published:** 2026-08-07

**Authors:** Jose A. Pinto-Tasende, Carlos Garcia-Porrua, Francisco Maceiras-Pan, Rafael B. Melero-González, Angeles Hernandez del Rio, Evelin Cecilia Cervantes Pérez, Victor Quevedo-Vila, Carlota L. Iñiguez, Sara Alonso-Castro, Maria Caeiro-Aguado, Rubén Queiro

**Affiliations:** 1 Department of Rheumatology-INIBIC, Complexo Hospitalario Universitario de A Coruña, 84 Xubias de Arriba Road, 15006 A Coruña, Spain; 2 Hospital Universitario Lucus Augusti, Rheumatology, 27003 Lugo, Spain; carlos.garcia.porrua@sergas.es; 3 Complexo Hospitalario Universitario Alvaro Cunqueiro, Rheumatology, 36312 Vigo, Spain; pacmaceiras99@yahoo.es; 4 Complexo Hospitalario Universitario Ourense, Rheumatology, 32005 Ourense, Spain; rafabmg@yahoo.es; 5 Complexo Hospitalario Universitario Arquitecto Marcide-Naval, Rheumatology, 15405 Ferrol, Spain; delrio8@yahoo.es; 6 Complexo Hospitalario Universitario Pontevedra, Rheumatology, 36071 Pontevedra, Spain; evelincervantesp@gmail.com (E.C.C.P.); maria.caeiro.aguado@sergas.es (M.C.-A.); 7 Hospital Comarcal Monforte de Lemos, Rheumatology, 27400 Lugo, Spain; victor.eliseo.quevedo.vila@sergas.es; 8 Hospital El Bierzo-Ponferrada, Rheumatology, 24404 León, Spain; carlotainiguezubiaga21@gmail.com; 9 Hospital Universitario Central de Asturias, Rheumatology, 33011 Oviedo, Spain; saraalonsocastro@hotmail.com (S.A.-C.); rubenque7@yahoo.es (R.Q.)

**Keywords:** psoriatic arthritis, bimekizumab, drug survival, treatment discontinuation, obesity, real-world evidence, biologic therapy, IL-17 inhibitors, effectiveness, persistence

## Abstract

**Background/Objectives:** Real-world evidence on bimekizumab treatment persistence and longitudinal disease activity in spondyloarthritis remains limited. This study assessed 12-month treatment persistence, changes in disease activity, and factors associated with discontinuation in patients with psoriatic arthritis and axial spondyloarthritis treated in routine clinical practice. **Methods:** We conducted a single-arm observational ambispective multicenter cohort study including 207 patients who initiated bimekizumab between December 2023 and November 2024. Treatment persistence was evaluated using Kaplan–Meier analysis and exploratory Cox proportional hazards models. Changes in disease activity were assessed using paired complete-case analyses, supplemented by available-case generalized estimating equation sensitivity analyses. **Results:** At 12 months, treatment persistence was 81.2%. Discontinuation occurred in 39 patients and was mainly attributed to lack of effectiveness. Persistence differed by sex in univariate analysis, with higher retention in males. Axial psoriatic arthritis classification was associated with an increased risk of discontinuation compared with peripheral psoriatic arthritis in the exploratory adjusted Cox model (HR: 3.02; 95% CI: 1.28–7.14). Obesity was not significantly associated with treatment discontinuation. Significant within-patient reductions were observed in BASDAI, ASDAS-CRP, and DAPSA among patients with available assessments. **Conclusions:** At 12 months, 81.2% of patients remained on treatment, and disease activity scores decreased among evaluable patients. Because this study had no comparator arm, these changes cannot be causally attributed to bimekizumab. The association between axial psoriatic arthritis classification and lower persistence should be considered hypothesis-generating because of the limited number of events, low event-to-parameter ratios, and absence of standardized axial PsA classification criteria. Safety data were restricted to adverse events leading to treatment discontinuation.

## 1. Introduction

Spondyloarthritis (SpA), encompassing axial spondyloarthritis (axSpA) and psoriatic arthritis (PsA), represents a heterogeneous group of chronic inflammatory diseases associated with substantial functional limitation and impaired quality of life. Although biologic and targeted synthetic therapies have expanded therapeutic options, a considerable proportion of patients do not achieve sustained disease control, underscoring the need for more effective and durable treatment strategies.

The interleukin (IL)-17 pathway plays a central role in the pathogenesis of SpA. Bimekizumab, a humanized monoclonal antibody that selectively neutralizes both IL-17A and IL-17F, has demonstrated significant efficacy across multiple randomized controlled trials in ankylosing spondylitis, non-radiographic axSpA, and PsA [1,2,3,4,5,6]. These studies consistently reported improvements in disease activity, physical function, and patient-reported outcomes, supporting the therapeutic relevance of dual IL-17A/F inhibition. However, randomized clinical trials are conducted under controlled conditions with strict eligibility criteria, which may limit their external validity and applicability to routine clinical practice [7].

Real-world evidence (RWE) complements randomized trials by evaluating treatment performance in broader and more heterogeneous populations, reflecting everyday clinical decision making and patient variability [8,9,10,11]. Within this framework, treatment persistence—commonly assessed through drug survival analyses—has emerged as a pragmatic endpoint that integrates effectiveness, safety, tolerability, and patient-related factors [12,13,14]. Persistence analyses using Kaplan–Meier methods and Cox proportional hazards models provide valuable insights into long-term treatment retention and determinants of discontinuation.

Although bimekizumab has shown robust efficacy in clinical trials, real-world data on its effectiveness and persistence in SpA remain limited. Furthermore, few studies have combined longitudinal assessments of disease activity with formal survival-based modeling. A better understanding of factors influencing treatment response and discontinuation in routine practice may help refine patient selection and optimize long-term outcomes. Potential sources of variability, including disease phenotype, sex, and metabolic factors, such as obesity, warrant further exploration [13,15,16,17,18,19,20].

Accordingly, this study aimed to describe 12-month treatment persistence and longitudinal changes in disease activity among patients with axial spondyloarthritis and psoriatic arthritis receiving bimekizumab in routine clinical practice. Specifically, we sought to (i) characterize longitudinal changes in disease activity, (ii) estimate 12-month treatment persistence using survival analysis, and (iii) explore clinical and demographic factors associated with treatment discontinuation.

## 2. Materials and Methods

### 2.1. Study Design and Population

We conducted an ambispective observational cohort study including consecutive patients who initiated treatment with 160 mg of bimekizumab between 1 December 2023 and 30 November 2024 at 9 participating centers. The database was locked on 1 December 2025. Follow-up was truncated at 12 months for all participants to provide a uniform risk window; therefore, this study was designed to estimate 12-month persistence rather than long-term drug survival. Patients were followed from treatment initiation until discontinuation, last available visit, or 12 months, whichever occurred first. The patients included were aged 18 years or over and met the CASPAR classification criteria for psoriatic arthritis [21], with moderate-to-high disease activity, as measured by DAPSA [22], despite treatment with conventional synthetic DMARDs (methotrexate, leflunomide or sulphasalazine), or with intolerance or contraindications, and they were considered candidates for biological therapy in accordance with recommendations for the management of PsA [23,24,25]. The axial SpA group included patients who met ASAS criteria [26] and started bimekizumab as their first biologic or following failure of a previous biologic, at the dose recommended in the summary of product characteristics. This study was conducted in accordance with the Declaration of Helsinki and was approved by the Ethics Committee of Santiago-Lugo (Protocol number 2015/671). Written informed consent was obtained from all subjects involved in this study.

This observational cohort study was reported in accordance with the Strengthening the Reporting of Observational Studies in Epidemiology (STROBE) statement; the completed STROBE checklist is provided as Supplementary Table S4.

The primary objective was to assess the 12-month treatment persistence of bimekizumab in patients with psoriatic arthritis and axial spondyloarthritis treated in routine clinical practice. Secondary objectives included evaluating longitudinal changes in disease activity using BASDAI, ASDAS-CRP, MDA, and DAPSA; describing reasons for treatment discontinuation; exploring clinical and demographic factors associated with discontinuation; and characterizing adverse events that led to permanent treatment withdrawal.

No a priori sample-size or power calculation was performed. The cohort comprised all consecutive eligible patients who initiated bimekizumab during the prespecified inclusion period at the nine participating centers; the sample size was, therefore, determined by the available real-world population rather than by a target effect estimate. Precision was summarized using 95% confidence intervals, and multivariable model complexity was constrained by the number of discontinuation events.

Clinical and demographic data were obtained from electronic medical records and prospective follow-up visits. Extra-musculoskeletal manifestations included uveitis and inflammatory bowel disease. The following comorbidities and lifestyle factors were recorded: smoking status, alcohol consumption, type 2 diabetes mellitus, obesity, dyslipidemia, hypertension, cardiovascular disease, hepatic steatosis, fibromyalgia, and depression. Obesity was defined as BMI ≥ 30 kg/m^2^ based on the recorded clinical assessment. Continuous BMI values and categorical obesity status were extracted separately when available. Comorbidities were identified through retrospective medical chart review and were based on previously documented specialist diagnoses.

Patients were reviewed in rheumatology clinics in accordance with standard clinical practice, and disease activity was assessed using joint counts, enthesitis and dactylitis, DAPSA and its cut-off points [27], BASDAI, and ASDAS-CRP at baseline, 6 months, 12 months, or the last assessment before discontinuation. MDA was evaluated in patients with peripheral or axial PsA when sufficient information was available to determine whether at least five of the seven MDA criteria were met [28]. Primary MDA summaries used observed cases. As a conservative sensitivity analysis, patients with a missing 12-month MDA assessment or treatment discontinuation before 12 months were classified as non-responders.

The index date was defined as the initiation of bimekizumab therapy. Patients were followed for up to 12 months when data were available. Treatment discontinuation was defined as permanent withdrawal of bimekizumab or interruption lasting ≥91 days, according to clinical records. Primary inefficacy was defined as lack of clinical response leading to treatment discontinuation within the first 6 months, whereas secondary inefficacy was defined as loss of response after initial improvement.

Adverse-event ascertainment was restricted to events documented as the reason for permanent bimekizumab discontinuation. Non-serious, transient, and non-discontinuation adverse events were not systematically collected. Therefore, incidence rates and the overall safety profile of bimekizumab could not be evaluated in this study.

### 2.2. Disease Phenotype Classification

Patients were classified into three phenotype groups: axial spondyloarthritis (axial SpA), peripheral psoriatic arthritis (peripheral PsA) without documented axial involvement, and axial psoriatic arthritis (axial PsA). Axial PsA was operationally defined as PsA fulfilling CASPAR criteria with axial involvement documented by the treating rheumatologist based on routine clinical assessment and/or available imaging of the sacroiliac joints or spine, with or without peripheral arthritis, dactylitis, or enthesitis. Imaging was not mandated by the study protocol, was not centrally reviewed, and the clinical-versus-imaging basis of classification was not captured as a structured variable. Because validated PsA-specific classification criteria for axial involvement were not available, this pragmatic phenotype assignment was considered exploratory [29,30].

In multivariable models, peripheral PsA was used as the reference category, and phenotypic clusters were entered as indicator variables. Two binary variables were, therefore, included in the models: axial SpA (yes/no) and axial PsA (yes/no).

### 2.3. Statistical Analysis

Descriptive statistics were used to summarize baseline characteristics. Continuous variables are presented as means ± standard deviation (SD) or medians and interquartile ranges (IQRs), depending on the distribution. Categorical variables are expressed as counts and percentages.

Comparisons across phenotypic clusters were performed using the Kruskal–Wallis test for continuous variables and the chi-square test for categorical variables. Post hoc pairwise comparisons were conducted using Mann–Whitney U tests with Bonferroni correction where appropriate.

Primary longitudinal analyses were restricted to patients with paired data at baseline and the relevant follow-up time point. Changes were assessed using the Wilcoxon signed-rank test.

As a sensitivity analysis, all available repeated measurements were additionally analyzed using generalized estimating equations with a Gaussian distribution, identity link, exchangeable working correlation structure, and robust standard errors, with time entered as a categorical variable.

Missing data were quantified for each variable and outcome. Baseline characteristics were compared between patients with and without available obesity status and, among patients with baseline ASDAS-CRP, between those with and without paired follow-up ASDAS-CRP. Multiple imputation was not performed because outcome recording was domain-specific and sparse, making the missing-at-random assumption difficult to justify. Complete-case datasets were used for the primary paired and regression analyses; the generalized estimating equation models served as available-case sensitivity analyses and did not eliminate the possibility of selection bias.

### 2.4. Survival Analysis

Time-to-event analyses were performed to evaluate treatment discontinuation over a 12-month follow-up period. The event was defined as permanent discontinuation of bimekizumab, while patients remaining on therapy were censored at their last available follow-up.

Kaplan–Meier methods were used to estimate treatment persistence, and survival curves were compared using the log-rank test.

Multivariable Cox proportional hazards models were constructed to explore factors associated with treatment discontinuation. The proportional hazards assumption was assessed using Schoenfeld residuals. Covariates included age, sex, disease phenotype, and disease duration, with an additional complete-case model including obesity status. Hazard ratios (HRs) and 95% confidence intervals (CIs) were reported. In a sensitivity analysis, biologic/targeted treatment line was modeled as a continuous variable rather than dichotomized as ≤2 versus ≥3.

The principal Cox model included 200 complete cases, 37 discontinuation events, and five estimated regression parameters, corresponding to 7.4 events per parameter. The extended model including obesity comprised 147 complete cases, 31 events, and six parameters, corresponding to 5.2 events per parameter. Both ratios were below the conventional benchmark of approximately 10 events per parameter, indicating a material risk of overfitting, unstable coefficients, imprecise confidence intervals, and optimistic statistical significance. Given these limitations and the lack of external validation, all multivariable analyses were considered exploratory.

During manuscript preparation and revision, ChatGPT v5 (OpenAI, San Francisco, CA, USA; accessed July 2026) was used to assist with language editing and with drafting and checking statistical code for the additional sensitivity analyses requested during peer review. The tool was not used to generate or modify primary data. All code, statistical outputs, scientific interpretations, and manuscript changes were reviewed and verified by the authors, who take full responsibility for the content.

## 3. Results

### 3.1. Patient Characteristics

A total of 207 patients initiated bimekizumab during the study period. The mean age was 54.3 ± 11.9 years, and 54.6% were female. The median disease duration was 9 years (IQR: 5–14).

The phenotypic distribution was peripheral PsA: 84 patients (40.6%); axial PsA: 65 patients (31.4%); and axial SpA: 58 patients (28.0%).

Baseline characteristics by phenotype are shown in Table 1, and characteristics for the overall cohort are provided in Supplementary Table S1. A continuous BMI value was available for 137 patients, whereas categorical obesity status was available for 149 patients.

### 3.2. Treatment Persistence

**Figure 1:**
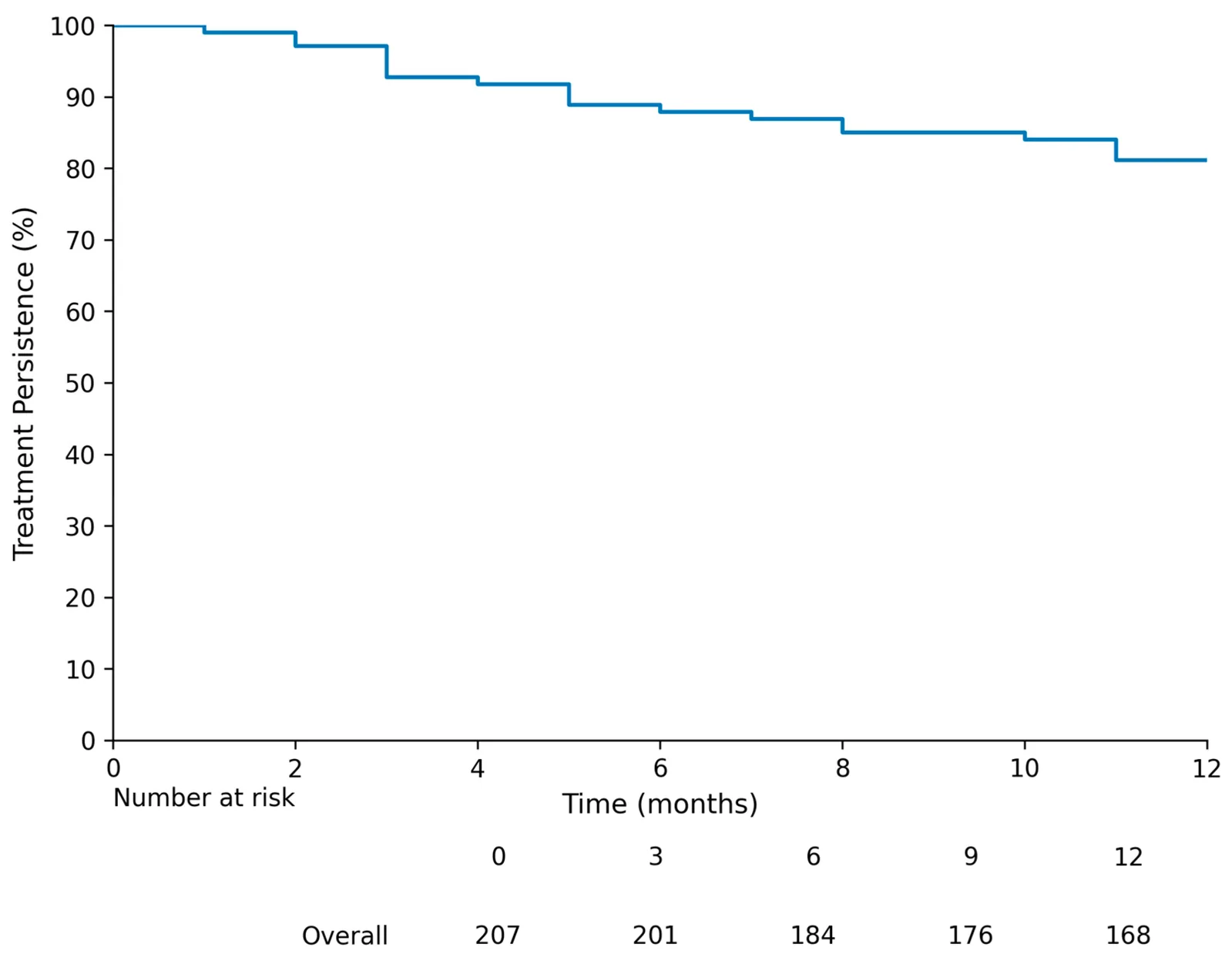
Overall treatment persistence over 12 months. Kaplan–Meier curve showing overall treatment persistence over 12 months. The event was defined as treatment discontinuation. Patients remaining on therapy at the end of follow-up were censored. Tick marks indicate censored observations.

At 12 months, treatment persistence was 81.2% (168/207). Kaplan–Meier analysis estimated a persistence probability of 0.812 at 12 months, indicating sustained treatment retention over time (Figure 1).

Treatment discontinuation was observed in 39 patients. Among patients with a documented reason for discontinuation (n = 36), lack of effectiveness accounted for 23 cases (63.9%): primary inefficacy in 18 patients (50.0%) and secondary loss of response in five patients (13.9%). Adverse events accounted for 13 documented discontinuations (36.1%). The reason for discontinuation was unavailable in three patients.

Persistence differed by sex (log-rank *p* = 0.039), with higher retention observed in males. No statistically significant differences were observed according to obesity status (log-rank *p* = 0.168).

#### 3.2.1. Persistence According to Patient Subgroups

##### Baseline Characteristics According to 12-Month Treatment Persistence

Baseline characteristics stratified by 12-month treatment persistence are presented in Table 2. Variable-specific denominators differed because several clinical variables were incompletely recorded. No significant differences were observed between patients who discontinued treatment and those who remained on therapy regarding age, body mass index (BMI), prior exposure to IL-17 inhibitors, previous biologic/targeted treatment line, early-line bimekizumab use, or number of comorbidities.

A higher proportion of obesity was observed among patients who discontinued treatment compared with those who maintained therapy (58.1% vs. 43.2%), although this difference did not reach statistical significance (*p* = 0.140). Overall, baseline demographic and clinical characteristics were broadly comparable between groups.

#### 3.2.2. Treatment Lines and Previous Treatment with IL-17A Inhibitors

**Figure 2:**
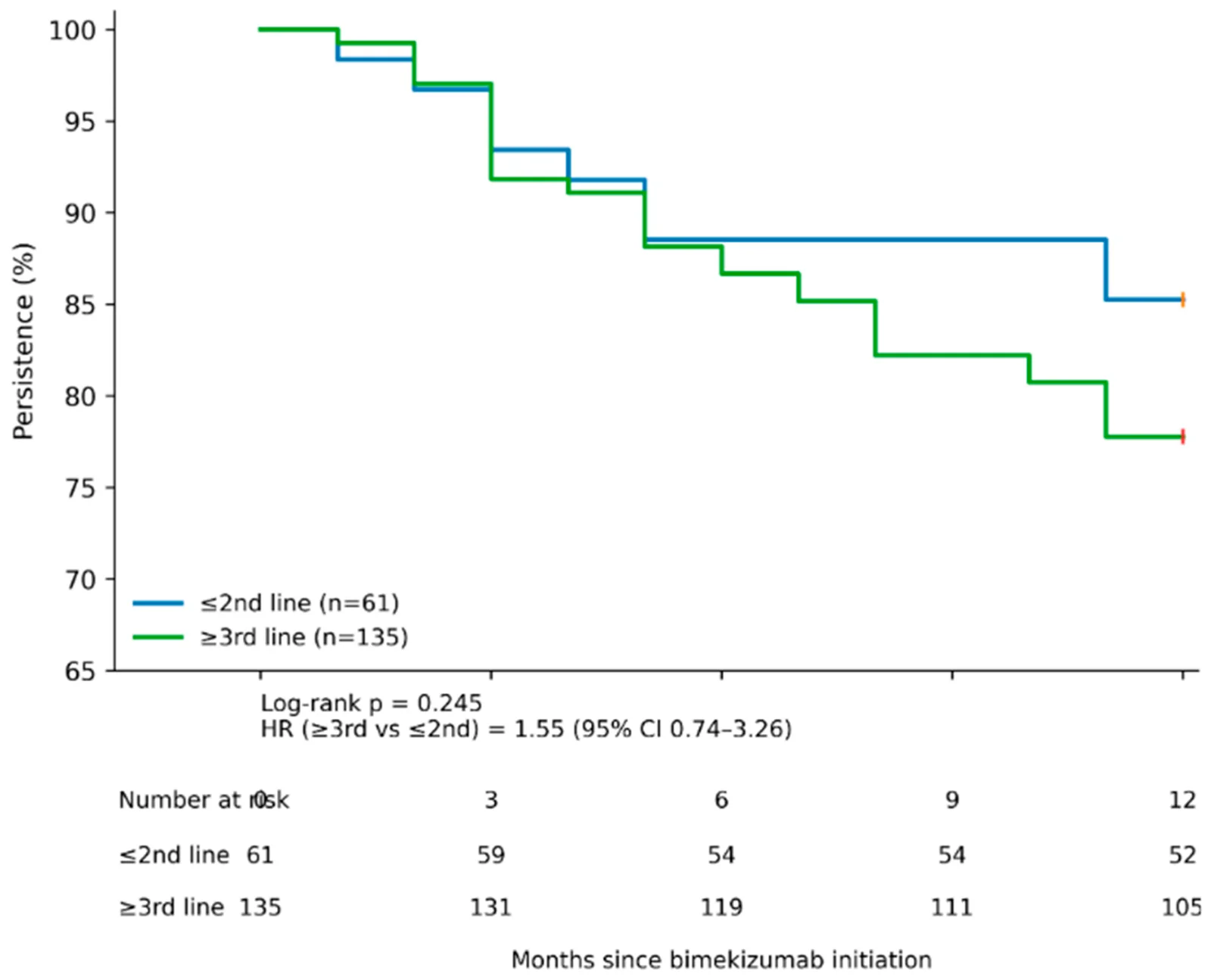
Kaplan–Meier treatment persistence at 12 months according to biologic treatment line. Patients were stratified according to the predefined variable BT ≤ 2 into earlier-line treatment (≤2nd biologic line) and later-line treatment (≥3rd biologic line). The event was permanent discontinuation of bimekizumab, and follow-up was truncated at 12 months. Tick marks indicate censored observations. The number at risk is shown below the plot. No significant differences were found regarding previous treatment with IL-17A inhibitors (*p* = 0.591).

Treatment persistence was numerically higher in patients receiving bimekizumab as ≤ second-line biologic/targeted therapy than in those treated in ≥third-line, although the difference was not statistically significant (12-month KM persistence: 85.2% [95% CI: 76.3–94.1] vs. 77.8% [95% CI: 70.8–84.8]; log-rank *p* = 0.245; HR: 1.55; 95% CI: 0.74–3.26; *p* = 0.250) (Figure 2). When the treatment line was modeled continuously, each one-line increase was not associated with discontinuation (unadjusted HR: 1.08; 95% CI: 0.93–1.26; *p* = 0.319; n = 196, 39 events). In an adjusted sensitivity model, the corresponding HR was 1.05 (95% CI: 0.87–1.26; *p* = 0.607; n = 191, 37 events).

#### 3.2.3. Disease Phenotype

**Figure 3:**
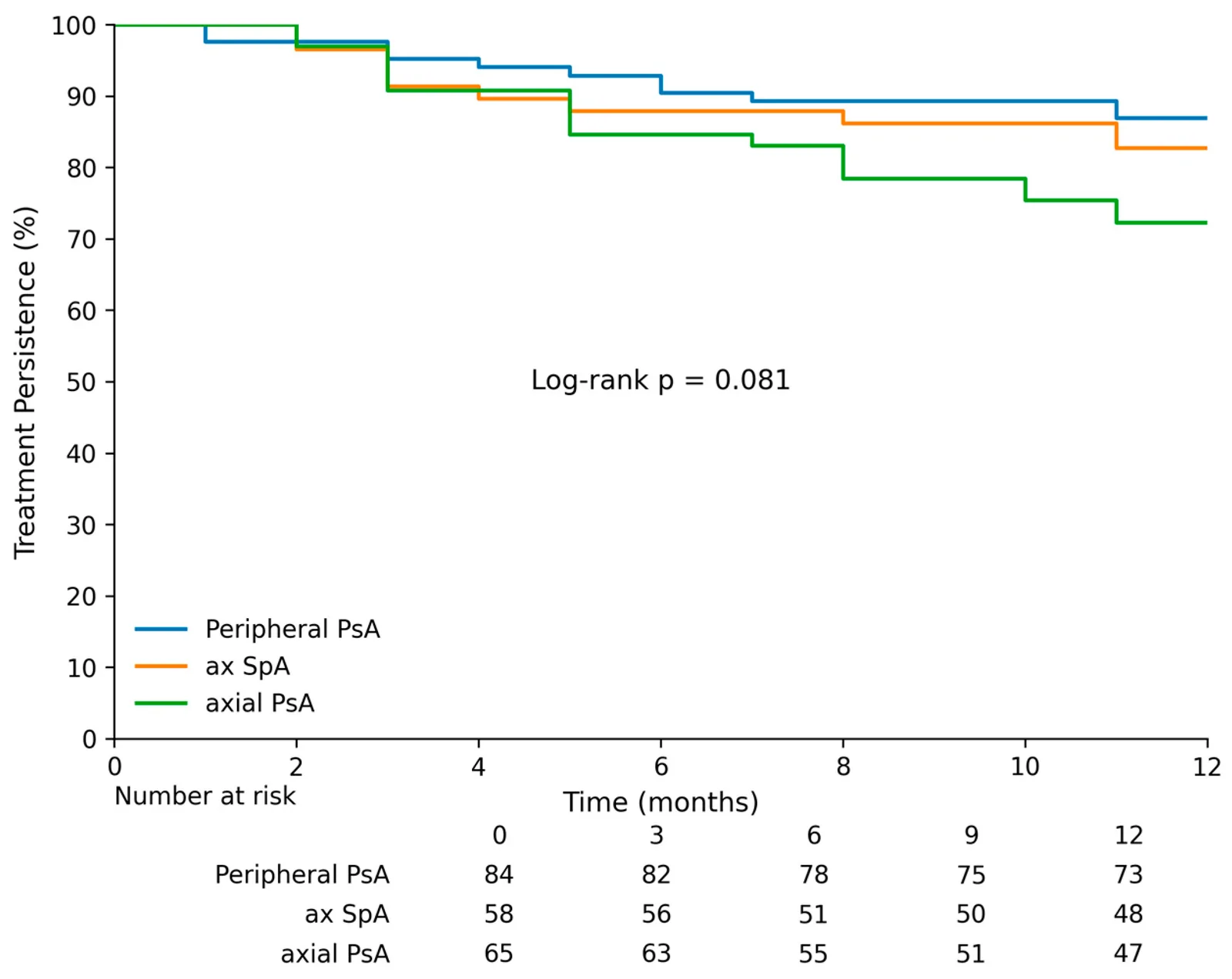
Treatment persistence by phenotypic cluster. Kaplan–Meier curves stratified by disease phenotype. The event was defined as treatment discontinuation, with censoring at the last follow-up visit. Group differences were evaluated using log-rank tests.

In the overall comparison across phenotypic groups, treatment persistence did not reach statistical significance (log-rank *p* = 0.081). However, exploratory pairwise comparisons suggested lower persistence in axial PsA compared with peripheral PsA (unadjusted *p* = 0.029) (Figure 3).

#### 3.2.4. Sex

**Figure 4:**
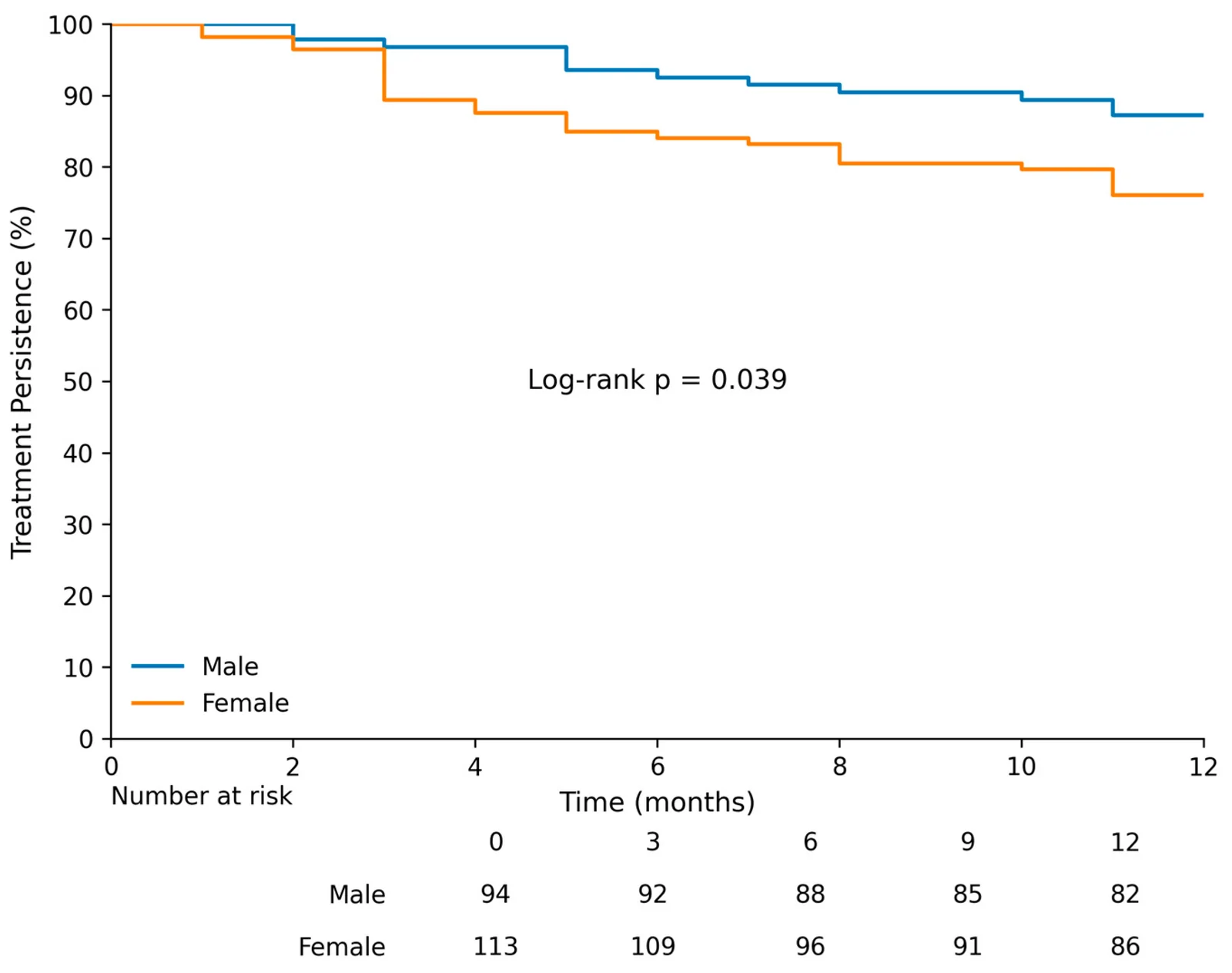
Treatment persistence by sex. Kaplan–Meier curves stratified by sex. Log-rank test compares groups.

Treatment persistence differed by sex (log-rank *p* = 0.039), with males demonstrating higher persistence over time compared with females (Figure 4).

#### 3.2.5. Obesity Status

**Figure 5:**
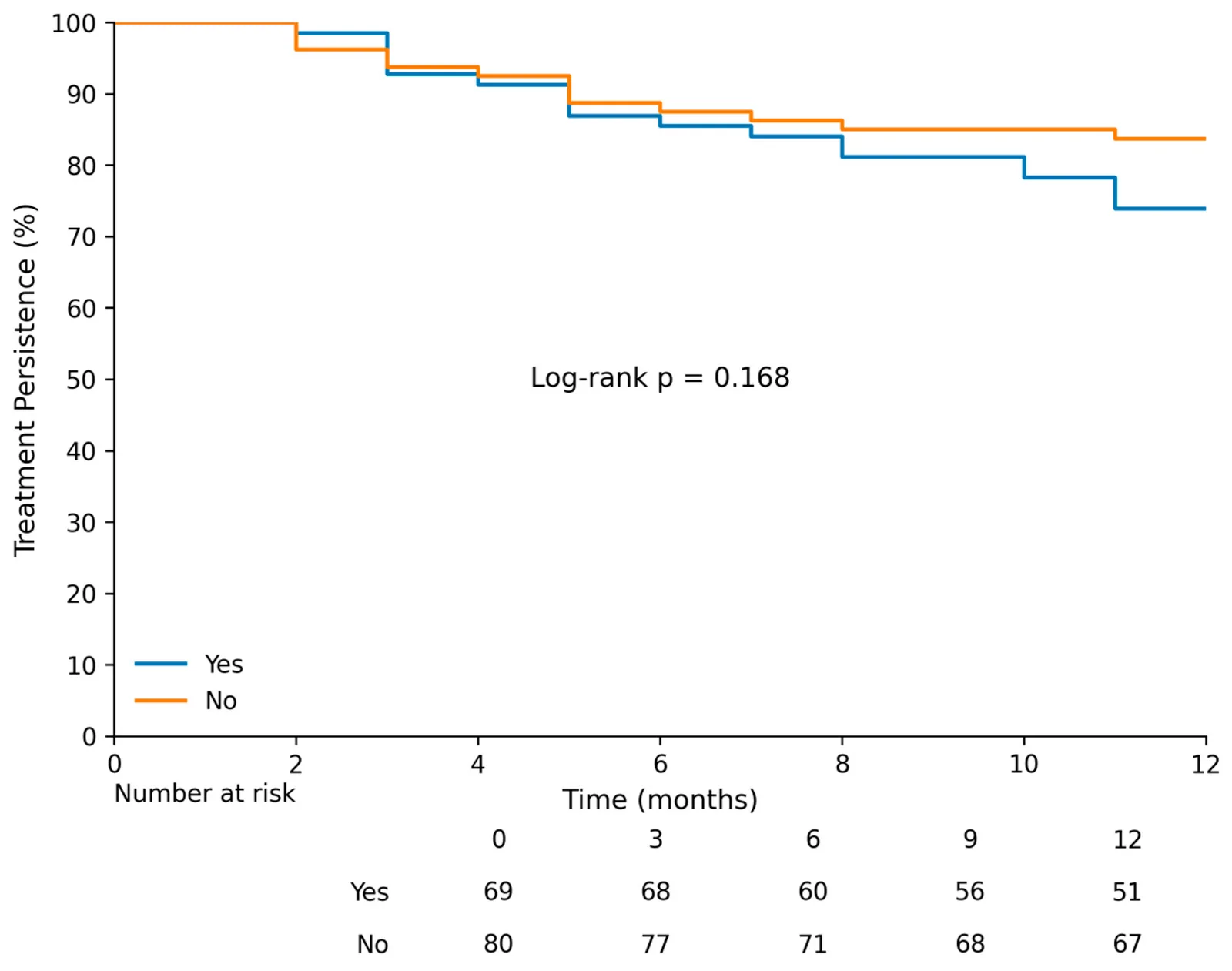
Treatment persistence stratified by obesity. Kaplan–Meier curves stratified by obesity status. Log-rank test compares groups.

Patients with obesity showed a numerically lower 12-month persistence rate compared with non-obese patients (73.9% vs. 83.8%), although this difference did not reach statistical significance (log-rank *p* = 0.168) (Figure 5).

### 3.3. Survival Analysis and Factors Associated with Discontinuation

In the principal complete-case Cox regression model adjusting for age, sex, disease phenotype, and disease duration, axial PsA classification was associated with a higher risk of discontinuation than peripheral PsA (HR: 2.38; 95% CI: 1.10–5.17; *p* = 0.028), whereas axial SpA was not significantly associated with discontinuation.

No significant associations were observed for age, sex, or disease duration.

In the extended complete-case model including obesity (Table 3), axial PsA classification remained associated with discontinuation (HR: 3.02; 95% CI: 1.28–7.14; *p* = 0.012), whereas obesity was not significantly associated with discontinuation (HR: 1.75; 95% CI: 0.84–3.66; *p* = 0.136).

No relevant violation of the proportional hazards assumption was detected. The modest event-to-parameter ratios (7.4 in the principal model and 5.2 in the obesity model) and the wide confidence intervals indicate limited precision; therefore, these results should be interpreted as exploratory associations rather than validated predictors.

**Figure 6:**
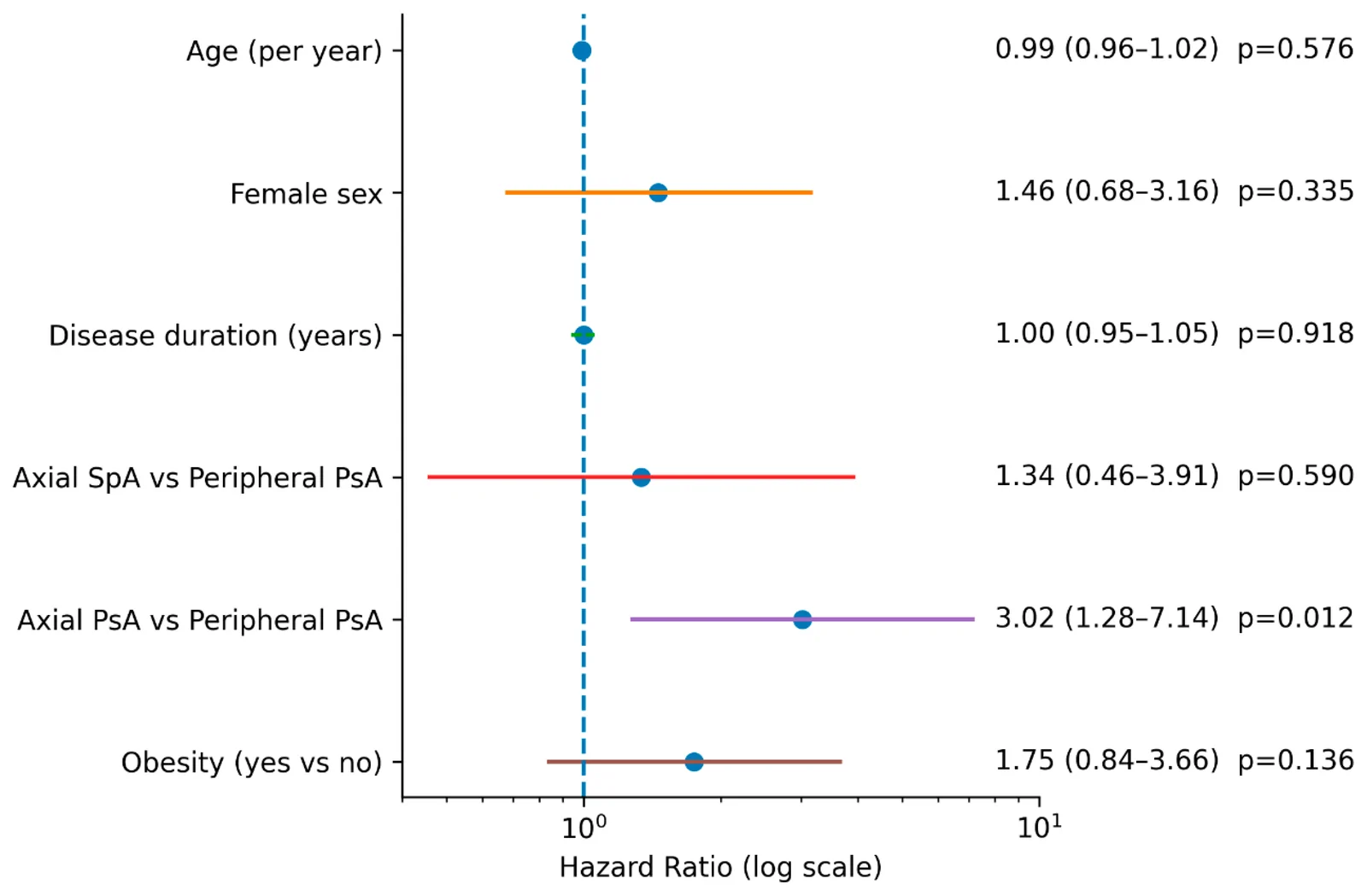
Forest plot of hazard ratios from the multivariable Cox proportional hazards model evaluating predictors of treatment discontinuation. Forest plot of hazard ratios from the multivariable Cox proportional hazards model evaluating predictors of treatment discontinuation. Hazard ratios (HRs) and 95% confidence intervals (CIs) are shown on a logarithmic scale. Peripheral PsA was used as the reference category for phenotypic cluster.

Axial PsA classification was the only covariate associated with treatment discontinuation in the exploratory extended model; no significant associations were observed for axial SpA, obesity, or the other covariates (Figure 6).

### 3.4. Disease Activity and Clinical Response

Significant improvements in disease activity were observed over time in patients with available paired data. The median BASDAI decreased from 6.00 (IQR: 4.72–7.45) at baseline to 3.41 (IQR: 2.00–5.90) at 6 months and 2.40 (IQR: 1.40–5.60) at 12 months (both *p* < 0.001). The median ASDAS-CRP decreased from 2.96 (IQR: 2.49–3.49) to 2.01 (IQR: 1.39–3.02) and 1.97 (IQR: 1.29–2.45), respectively (*p* < 0.001 and *p* = 0.002). The median DAPSA decreased from 23.12 (IQR: 18.50–30.00 to 8.40 (IQR: 4.40–14.00) and 6.10 (IQR: 2.08–9.75), respectively (both *p* < 0.001). The available-case generalized estimating equation sensitivity analyses were directionally consistent: the estimated mean changes at 6 and 12 months were −2.00 (95% CI: −2.71 to −1.29) and −2.67 (95% CI: −3.39 to −1.96) for BASDAI, −1.05 (95% CI: −1.45 to −0.66) and −1.11 (95% CI: −1.51 to −0.70) for ASDAS-CRP, and −13.23 (95% CI: −15.27 to −11.19) and −17.90 (95% CI: −20.22 to −15.59) for DAPSA (all *p* < 0.001).

The observed-case MDA rates at 12 months were 97.0% (64/66) in peripheral PsA and 92.2% (47/51) in axial PsA. These estimates were based on patients with evaluable MDA data and were strongly influenced by complete-case and survivor selection: among PsA patients who persisted to 12 months, 108/109 (99.1%) of those with evaluable data met MDA, whereas only 8/29 discontinuers had an evaluable MDA assessment. In a conservative non-responder imputation sensitivity analysis using the complete baseline phenotype denominators, the 12-month MDA rates were 76.2% (64/84) and 72.3% (47/65), respectively (*p* = 0.590). The corresponding median DAPSA values of 5.00 and 6.55 were within the low-disease-activity range, rather than the moderate range. No significant differences in persistence or disease-activity outcomes were consistently observed across phenotype groups (Table 4).

Significant improvements in disease activity were observed over time in patients with available paired data. At baseline, most patients had high or very high disease activity, as defined by the ASDAS criteria. At 6 months, 16.7% achieved inactive disease, and 40.0% achieved low disease activity. At 12 months, 24.0% achieved inactive disease, and 24.0% achieved low disease activity. The proportion of patients with very high disease activity decreased substantially over time (Supplementary Figure S1).

Regarding ASDAS improvement, 46.4% of patients achieved clinically important improvement, and 17.9% achieved major improvement at 6 months. At 12 months, 45.8% achieved clinically important improvement, and 8.3% achieved major improvement. Because only 28 and 24 patients had paired ASDAS-CRP data at 6 and 12 months, respectively, the previously reported multivariable model for clinical response was removed to avoid unstable and overinterpreted estimates.

These results are summarized in Table 5 and illustrated in Supplementary Table S2 and Supplementary Figures S1 and S2. Missing-data comparisons and sensitivity analysis are provided in Supplementary Tables S5–S9.

#### Missing-Data and Sensitivity Analyses

Among the 48 patients with baseline ASDAS-CRP, 28 had paired 6-month data, and 24 had paired 12-month data. Patients with and without paired follow-up ASDAS-CRP did not differ significantly in age, sex, phenotype, disease duration, treatment line, obesity status, persistence, or baseline ASDAS-CRP (all *p* > 0.05), although baseline ASDAS-CRP tended to be lower among patients with available 12-month data (median: 2.89 vs. 3.35; *p* = 0.074). These comparisons had limited power and do not exclude selection bias.

Obesity status was available for 149 patients and missing for 58. Compared with patients without recorded obesity status, those with available obesity data had a higher median biologic/targeted treatment line (4.0 vs. 3.0; *p* = 0.003), more frequent prior IL-17 inhibitor exposure (38.5% vs. 23.2%; *p* = 0.048), and higher comorbidity count (*p* = 0.008). Age, sex, phenotype distribution, disease duration, and 12-month persistence did not differ significantly. These findings support cautious interpretation of the obesity complete-case analysis.

No multivariable predictive model for clinical response was retained in the revised manuscript. The available-case generalized estimating equation analyses confirmed the direction and statistical significance of the paired longitudinal findings but do not correct for potentially informative missingness.

### 3.5. Adverse Events Leading to Discontinuation

Among the adverse events documented as leading to discontinuation, infectious and mucocutaneous events, particularly candidiasis, were the most frequent. Other events were less common and included systemic and immune-mediated conditions. These data describe only adverse events leading to discontinuation, resulting in permanent withdrawal, and should not be interpreted as a comprehensive safety analysis. Details are provided in Supplementary Table S3.

## 4. Discussion

This ambispective single-arm real-world study estimated 12-month treatment persistence and described longitudinal changes in disease activity among patients with spondyloarthritis receiving bimekizumab. The observed 12-month persistence rate of 81.2% indicates substantial first-year treatment retention and is consistent with reports of biologic drug survival in inflammatory rheumatic diseases [13,14,15]. Persistence is a composite endpoint integrating effectiveness, tolerability, safety, access, and patient- and clinician-related factors [12,13,14].

Treatment discontinuation was primarily attributed to lack of effectiveness. Among patients with a documented reason, lack of effectiveness accounted for 63.9% and adverse events for 36.1%. However, adverse-event ascertainment was restricted to events leading to permanent withdrawal. This study cannot, therefore, estimate the overall incidence of adverse events or compare its safety profile with randomized trials or other real-world cohorts.

The global comparison across phenotype groups was not statistically significant, but pairwise and exploratory adjusted analyses suggested lower persistence in patients classified as having axial PsA than in peripheral PsA. This finding requires particular caution because axial PsA was assigned pragmatically in routine practice, without protocol-mandated imaging, central image review, or validated PsA-specific classification criteria. The international AXIS initiative is developing and validating standardized criteria [29,30]; until such criteria are available, the present result should be regarded as hypothesis-generating.

Sex-related differences were observed in the unadjusted analysis but were not confirmed in the adjusted model. Obesity was not significantly associated with persistence; however, obesity status was missing in 58 patients. Patients with available obesity data had later treatment lines, more frequent previous IL-17 inhibitor exposure, and a higher comorbidity burden, indicating that the complete-case obesity analysis may be affected by selection.

The very high observed-case MDA rates at 12 months warrant careful interpretation. They were calculated only among patients with evaluable MDA data and were strongly enriched for patients who remained on treatment. A conservative non-responder imputation analysis reduced the estimates to 76.2% in peripheral PsA and 72.3% in axial PsA. Moreover, the 12-month DAPSA medians were in the low-disease-activity range, making the direction of the MDA results clinically coherent, although MDA and DAPSA assess partially different domains and may be discordant in individual patients.

Baseline characteristics were broadly comparable between patients who discontinued and those who persisted, with a non-significant numerical excess of obesity among discontinuers. Because categorical obesity status and continuous BMI were available in only 149 and 137 patients, respectively, the absence of a statistically significant association should not be interpreted as evidence of no effect.

In the exploratory Cox analyses, axial PsA classification was associated with discontinuation, whereas obesity, sex, age, and disease duration were not. The modest event-to-parameter ratios, wide confidence intervals, non-standardized phenotype definition, and absence of external validation preclude describing these variables as established predictors. The results identify associations that require confirmation in larger prospective cohorts.

Treatment line was examined as a possible marker of refractory disease. The original comparison between the ≤ second and ≥ third line was not statistically significant. Modeling treatment line continuously produced the same conclusion, with no association between each additional line and discontinuation in either the unadjusted or adjusted sensitivity analysis.

Substantial within-patient reductions in BASDAI, ASDAS-CRP, and DAPSA were observed in paired analyses and were supported by available-case generalized estimating equation sensitivity analyses. However, this was a single-arm cohort without an untreated or active comparator; consequently, these temporal changes cannot be causally attributed to bimekizumab. Regression to the mean, observation effects, concurrent changes in comorbidity or symptomatic management, and other time-varying influences remain plausible alternative or contributing explanations. In addition, the small paired samples—particularly for ASDAS-CRP—limit precision and generalizability. These analyses describe changes among patients with recorded assessments rather than a causal treatment effect in the entire cohort.

Among recorded adverse events leading to discontinuation, candidiasis and other mucocutaneous events were consistent with events previously associated with IL-17A/F inhibition. Because non-discontinuation adverse events were not systematically collected, no conclusion can be made regarding the overall safety profile or the absence of uncommon or delayed safety signals.

The missing-data analysis did not identify statistically significant baseline differences between patients with and without paired ASDAS-CRP follow-up, although the comparisons were underpowered and baseline ASDAS-CRP tended to be lower among those with 12-month data. Multiple imputation was not considered sufficiently defensible because measurement was phenotype- and domain-dependent and the missing-at-random assumption was uncertain. The available-case sensitivity models used more observations but do not eliminate potential informative missingness.

The observational design also precludes causal interpretation of factors associated with discontinuation. Residual confounding may arise from unmeasured disease severity, adherence, patient preferences, expectations, access to alternative therapies, and physician-specific discontinuation practices. Propensity-score methods were not applied because all participants received bimekizumab, phenotype was not a treatment assignment, the number of discontinuation events was limited, and propensity methods would not address unmeasured confounding.

This study has several limitations. It was a single-arm cohort without a comparator, so observed reductions in disease activity scores cannot be interpreted as causal treatment effects. Follow-up was restricted to 12 months; longitudinal outcome data were incomplete; obesity and BMI data were missing for a substantial proportion of patients; only adverse events leading to withdrawal were systematically ascertained; axial PsA was classified using a pragmatic non-standardized definition with potential misclassification; and the number of discontinuation events produced event-to-parameter ratios below the conventional 10-per-parameter benchmark. The resulting risk of overfitting, unstable coefficients, and imprecise estimates is a major limitation of the Cox models. The Cox and subgroup analyses should, therefore, be interpreted as exploratory. Larger prospective comparative cohorts with protocolized phenotype classification, standardized outcome collection, comprehensive safety surveillance, and longer follow-up are needed.

Despite these limitations, this study provides a multicenter real-world estimate of first-year bimekizumab persistence and documents improvement across peripheral and axial disease activity measures. The findings support further evaluation of bimekizumab in routine practice while identifying methodological priorities for subsequent studies.

## 5. Conclusions

In this single-arm real-world multicenter cohort, 81.2% of patients remained on bimekizumab at 12 months, and disease activity scores decreased among patients with available longitudinal assessments. These within-patient changes cannot be interpreted as causal treatment effects because no comparator arm was included. Discontinuation was more frequently attributed to insufficient effectiveness than to adverse events. Axial PsA classification was associated with lower persistence in exploratory adjusted analyses, but this finding requires external validation using standardized phenotype criteria, larger event numbers, and more stable multivariable models. Obesity was not significantly associated with discontinuation, although missing data limit inference. Because safety ascertainment was restricted to events causing permanent withdrawal, no conclusions regarding the overall safety profile can be drawn.

## Figures and Tables

**Table 1:** Baseline characteristics of the study cohort.

Variable	Peripheral PsA (n = 84)	Axial PsA (n = 65)	Ax SpA (n = 58)	*p*-Value
Age, years	54.17 ± 12.39	56.42 ± 11.88	51.93 ± 10.69	0.122
Female sex	49 (58.3%)	37 (56.9%)	27 (46.6%)	0.345
Disease duration, years	9 (5–14.2)	9 (5–14)	7.5 (4–12)	0.868
BMI, kg/m^2^	30.92 ± 7.01	30.88 ± 8.17	29.71 ± 6.22	0.683
CRP, mg/dL	0.60 (0.20–3.05)	0.91 (0.28–3.00)	0.41 (0.20–2.97)	0.370
DAPSA baseline	22.10 (18.00–28.01)	23.12 (18.04–30.38)	–	0.628
ASDAS-CRP baseline	–	2.80 (2.50–3.20)	3.30 (2.55–4.25)	0.203

Data are presented as means ± SD or medians (IQR) as appropriate. Comparisons were performed using Kruskal–Wallis test for continuous variables and the chi-square test for categorical variables. *p*-values < 0.05 are considered statistically significant.

**Table 2:** Baseline characteristics according to 12-month treatment persistence.

Variable	Discontinued (n = 39)	Persisted/Censored (n = 168)	*p*-Value
Age, years	54.6 ± 9.3 (n = 39)	54.2 ± 12.4 (n = 168)	0.798
BMI, kg/m^2^	29.8 ± 4.9 (n = 30)	30.8 ± 7.7 (n = 107)	0.370
Obesity	18/31 (58.1%)	51/118 (43.2%)	0.140
Previous biologic/targeted line, n	3.00 (3.00–5.00) (n = 39)	3.00 (2.00–4.00) (n = 157)	0.294
Bimekizumab as line ≤ 2	9/39 (23.1%)	52/157 (33.1%)	0.225
Prior IL-17 inhibitor exposure	12/39 (30.8%)	58/165 (35.2%)	0.604
Comorbidities, n	1.00 (1.00–3.00) (n = 39)	1.00 (0.00–2.00) (n = 163)	0.233

Data are presented as means ± standard deviation (n), medians (interquartile range) (n), or n/N (%), as appropriate. Between-group comparisons used Welch’s *t*-test, Mann–Whitney U test, chi-square test, or Fisher’s exact test, as appropriate. Variable-specific denominators reflect available-case data: continuous BMI, n = 137; categorical obesity status, n = 149; biologic/targeted treatment line and early-line classification, n = 196; prior IL-17 inhibitor exposure, n = 204; and comorbidity count, n = 202. No imputation was performed.

**Table 3:** Exploratory multivariable Cox proportional hazards model for treatment discontinuation.

Variable	HR (95% CI)	*p*-Value
Age (per year)	0.99 (0.96–1.02)	0.576
Female sex	1.46 (0.68–3.16)	0.335
Disease duration (years)	1.00 (0.95–1.05)	0.918
Axial SpA vs. peripheral PsA	1.34 (0.46–3.91)	0.590
Axial PsA vs. peripheral PsA	3.02 (1.28–7.14)	0.012
Obesity (yes vs. no)	1.75 (0.84–3.66)	0.136

Peripheral PsA was used as the reference category.

**Table 4:** Clinical outcomes by phenotypic cluster.

Variable	Peripheral PsA (n = 84)	Axial SpA (n = 58)	Axial PsA (n = 65)	*p*-Value
Persistence at 12 months	73/84 (86.9%)	48/58 (82.8%)	47/65 (72.3%)	0.073
MDA at 6 months (observed cases)	68/81 (84.0%)	–	46/65 (70.8%)	0.056
MDA at 12 months (observed cases)	64/66 (97.0%)	–	47/51 (92.2%)	0.242
DAPSA at 6 months	10.00 (4.33–16.50) (n = 51)	–	10.70 (6.00–14.50) (n = 40)	0.583
DAPSA at 12 months	5.00 (1.75–8.18) (n = 28)	–	6.55 (3.25–10.75) (n = 26)	0.088
ASDAS-CRP at 6 months	–	2.44 (1.75–3.25) (n = 18)	1.55 (1.29–2.11) (n = 12)	0.059
ASDAS-CRP at 12 months	–	2.30 (1.48–2.73) (n = 15)	1.73 (1.29–2.12) (n = 10)	0.437
BASDAI at 6 months	–	4.50 (3.00–7.15) (n = 18)	2.00 (1.55–4.38) (n = 20)	0.005
BASDAI at 12 months	–	2.90 (1.80–5.80) (n = 13)	2.00 (1.00–4.14) (n = 12)	0.149

Data are presented as n/N (%) or medians (IQR) (n). MDA rows in Table 4 use observed cases; conservative non-responder imputation results are reported in the text and Supplementary Table S8. MDA and DAPSA comparisons include PsA phenotypes only. BASDAI and ASDAS-CRP comparisons include axial-disease phenotypes only. Comparisons used the chi-square test and the Kruskal–Wallis test, as appropriate.

**Table 5:** Changes in BASDAI and ASDAS over time.

Outcome	Baseline, Median (IQR)	Follow-Up, Median (IQR)	Paired n	*p*-Value
BASDAI: baseline vs. 6 months	6.00 (4.72–7.45)	3.41 (2.00–5.90)	38	<0.001
BASDAI: baseline vs. 12 months	6.00 (4.80–7.55)	2.40 (1.40–5.60)	25	<0.001
ASDAS-CRP: baseline vs. 6 months	2.96 (2.49–3.49)	2.01 (1.39–3.02)	28	<0.001
ASDAS-CRP: baseline vs. 12 months	2.89 (2.44–3.40)	1.97 (1.29–2.45)	24	0.002
DAPSA: baseline vs. 6 months	23.12 (18.50–30.00)	8.40 (4.40–14.00)	65	<0.001
DAPSA: baseline vs. 12 months	23.00 (18.94–27.91)	6.10 (2.08–9.75)	42	<0.001

Values are expressed as medians (interquartile range). Wilcoxon signed-rank test. Each row uses the paired subset available for that specific baseline–follow-up comparison.

## Data Availability

The data are available from the corresponding author upon request.

## Supplementary Materials

The following supporting information can be downloaded at https://www.mdpi.com/article/10.3390/biomedicines14081778/s1.

## Abbreviations

ASAS	Assessment of Spondyloarthritis International Society
ASDAS	Ankylosing Spondylitis Disease Activity Score
ASDAS-CRP	Ankylosing spondylitis disease activity score using C-reactive protein
AXIS	Axial involvement in psoriatic arthritis
axSpA	Axial spondyloarthritis
BASDAI	bath ankylosing spondylitis disease activity index
BMI	Body mass index
CASPAR	Classification Criteria for Psoriatic Arthritis
CI	Confidence interval
CRP	C-reactive protein
DAPSA	Disease activity index for psoriatic arthritis
DMARDs	Disease-modifying antirheumatic drugs
GenAI	Generative artificial intelligence
HLA-B27	Human leukocyte antigen B27
HR	Hazard ratio
IL	Interleukin
IL-17	Interleukin-17
IL-17A	Interleukin-17A
IL-17F	Interleukin-17F
IL-17A/F	Interleukin-17A and interleukin-17F
IQR	Interquartile range
KM	Kaplan–Meier
MDA	Minimal disease activity
OR	Odds ratio
PsA	Psoriatic arthritis
RWE	Real-world evidence
SD	Standard deviation
SpA	Spondyloarthritis
STROBE	Strengthening the Reporting of Observational Studies in Epidemiology
