# Supplementary material for: Treatment Persistence and Factors Associated with Bimekizumab Discontinuation in Psoriatic Arthritis and Axial Spondyloarthritis: A Real-World Multicenter Study
Source: Biomedicines. 2026 Aug 7;14(8):1778. doi: 10.3390/biomedicines14081778 (PMC13509830; doi:10.3390/biomedicines14081778)
Supplement: Supplementary file 1 [file biomedicines-14-01778-s001.zip › biomedicines-4447713-supplementary.pdf]

Additional Supplementary Tables and Figures

Treatment Persistence and Factors Associated with Bimekizumab Discontinuation in Psoriatic Arthritis and Axial Spondyloarthritis: A Real-World Multicenter Study

Supplementary Table S1. Baseline characteristics of the study cohort

Characteristic	Total cohort (n = 207)
Age, years	54.2 ± 11.8
Female sex, n (%)	113 (54.6%)
Male sex. n (%)	94 (45.4%)
BMI, kg/m²	30.6 ± 7.2
Obesity, n (%)	69 (46.3%)
Disease duration, years	9 (5–14)
Phenotypic cluster: Axial SpA	58 (28.0%)
Phenotypic cluster: Axial PsA	65 (31.4%)
Phenotypic cluster: Peripheral PsA	84 (40.6%)
HLA-B27 positive	51 (24.6%)
Current smoker	45 (25.6%)
Former smoker	27 (15.3%)
Never smoker	104 (59.1%)

Data are presented as mean ± SD (n), median (IQR) (n), or n/N (%). For obesity and smoke status, the corrected denominator is the number of patients with available data (N = 149 and N = 176, respectively).

Supplementary Table S2. Predictors of ASDAS clinically important improvement*

Variable	Odds Ratio (OR)	95% CI	p-value
Age	1.01	0.97–1.05	0.610
Female sex	1.72	0.62–4.77	0.302
BMI	0.98	0.91–1.05	0.544
HLA-B27 positive	1.21	0.40–3.67	0.735
Disease duration	0.96	0.89–1.03	0.228
Baseline ASDAS	2.48	1.12–5.52	0.025

** Outcome: ASDAS clinically important improvement, defined as ΔASDAS ≥ 1.1.*

Supplementary Table S3. Adverse events leading to treatment discontinuation, grouped by category

Category	Adverse event	n/N (%)
Infectious	Refractory oropharyngeal candidiasis	3/13 (23.1%)
Infectious	Cutaneous lesions, suspected mycosis	1/13 (7.7%)
Mucocutaneous	Severe stomatitis	1/13 (7.7%)
Mucocutaneous	Urticarial reaction	1/13 (7.7%)
Mucocutaneous	Photosensitivity	1/13 (7.7%)
Systemic	Intolerance	1/13 (7.7%)
Immune-mediated	Inflammatory bowel disease	1/13 (7.7%)
Serious adverse event	Breast cancer	1/13 (7.7%)
Unspecified	Unspecified adverse event leading to discontinuation	3/13 (23.1%)

Percentages use all adverse-event discontinuations as denominator (N = 13). Three adverse-event discontinuations had no specific event recorded in the dataset and are therefore shown as unspecified.

Supplementary Table S4. STROBE checklist for observational cohort studies

Item	Recommendation
1a	Indicate the study's design with a commonly used term in the title or abstract.
1b	Provide in the abstract an informative and balanced summary of what was done and what was found.
2	Explain the scientific background and rationale for the investigation.
3	State specific objectives, including any prespecified hypotheses.
4	Present key elements of study design early in the paper.
5	Describe the setting, locations, and relevant dates, including periods of recruitment, exposure, and follow-up.
6a	Give eligibility criteria and participant selection methods.
7	Clearly define outcomes, exposures, predictors, confounders, and effect modifiers.
8	Describe data sources and measurement methods.

9	Describe efforts to address potential sources of bias.
10	Explain how the study size was arrived at.
11	Explain how quantitative variables were handled.
12	Describe statistical methods, including confounding control, subgroup analyses, missing data, and sensitivity analyses.
13	Report numbers of individuals at each stage; consider a flow diagram.
14	Give participant characteristics and indicate missing data.
15	Report outcome events or summary measures over time.
16	Provide effect estimates with precision, e.g., 95% CIs.
17	Report other analyses, including subgroups and interactions.
18	Summarize key results with reference to study objectives.
19	Discuss limitations, including potential sources of bias and imprecision.
20	Provide cautious interpretation considering objectives, limitations, and multiplicity.
21	Discuss generalizability/external validity.
22	Give source of funding and role of funders.

Supplementary Table S5. Baseline characteristics according to availability of categorical obesity status.

Variable	Obesity status available (n=149)	Obesity status unavailable (n=58)	p-value
Age, years	54.4 ± 12.2	53.9 ± 10.9	0.770
Female sex	82/149 (55.0%)	31/58 (53.4%)	0.877
Peripheral PsA	59/149 (39.6%)	25/58 (43.1%)	
Axial PsA	49/149 (32.9%)	16/58 (27.6%)	0.760
Axial SpA	41/149 (27.5%)	17/58 (29.3%)	
Disease duration, years	8.0 (5.0–13.0) (n=147)	10.0 (4.0–14.0) (n=53)	0.491
Biologic/targeted treatment line	4.0 (2.0–5.0) (n=148)	3.0 (2.0–3.25) (n=48)	0.003
Prior IL-17 inhibitor exposure	57/148 (38.5%)	13/56 (23.2%)	0.048
Comorbidities, n	1.0 (0.0–3.0) (n=146)	1.0 (0.0–1.25) (n=56)	0.008
Persistence at 12 months	118/149 (79.2%)	50/58 (86.2%)	0.323

Data are mean ± SD, median (IQR), or n/N (%). Continuous BMI values were available for 137 patients; categorical obesity status used in the Cox model was available for 149. P-values used Welch's t-test, Mann–Whitney U test, Fisher's exact test, or chi-square test, as appropriate.

Supplementary Table S6A. Baseline characteristics among patients with baseline ASDAS-CRP according to availability of paired 6-month ASDAS-CRP.

Variable	Paired 6-month ASDAS (n=28)	No paired 6-month ASDAS (n=20)	p-value
Age, years	53.2 ± 14.5	51.8 ± 8.9	0.671
Female sex	11/28 (39.3%)	9/20 (45.0%)	0.771
Axial PsA phenotype	11/28 (39.3%)	6/20 (30.0%)	0.555
Disease duration, years	9.0 (4.0–20.0)	7.0 (5.0–13.5) (n=19)	0.736
Biologic/targeted treatment line	4.0 (2.5–5.5) (n=27)	3.0 (2.5–4.0) (n=19)	0.084
Obesity	7/23 (30.4%)	7/15 (46.7%)	0.492
Baseline ASDAS-CRP	2.96 (2.49–3.49)	3.24 (2.52–4.33)	0.510
Persistence at 12 months	27/28 (96.4%)	17/20 (85.0%)	0.294

Data are mean ± SD, median (IQR), or n/N (%). Comparisons are exploratory and underpowered.

Supplementary Table S6B. Baseline characteristics among patients with baseline ASDAS-CRP according to availability of paired 12-month ASDAS-CRP.

Variable	Paired 12-month ASDAS (n=24)	No paired 12-month ASDAS (n=24)	p-value
Age, years	52.7 ± 13.4	52.6 ± 11.6	0.973
Female sex	9/24 (37.5%)	11/24 (45.8%)	0.770
Axial PsA phenotype	10/24 (41.7%)	7/24 (29.2%)	0.547
Disease duration, years	10.0 (4.0–20.0)	6.0 (5.0–12.5) (n=23)	0.382
Biologic/targeted treatment line	4.0 (2.5–5.5) (n=23)	3.0 (2.5–4.0) (n=23)	0.377
Obesity	5/19 (26.3%)	9/19 (47.4%)	0.313
Baseline ASDAS-CRP	2.89 (2.44–3.40)	3.35 (2.77–4.43)	0.074
Persistence at 12 months	23/24 (95.8%)	21/24 (87.5%)	0.609

Data are mean ± SD, median (IQR), or n/N (%). Comparisons are exploratory and underpowered.

Supplementary Table S7. Sensitivity analyses for biologic/targeted treatment line.

Model	n	Events	HR (95% CI)	p-value
Dichotomous: ≥3 vs. ≤2 line, unadjusted	196	39	1.55 (0.74–3.26)	0.250
Continuous: per one-line increase, unadjusted	196	39	1.08 (0.93–1.26)	0.319
Continuous: per one-line increase, adjusted*	191	37	1.05 (0.87–1.26)	0.607

**Adjusted for age, sex, disease duration, and disease phenotype. The dichotomous estimate is the original reported comparison.*

Supplementary Table S8. Minimal disease activity observed-case and non-responder-imputation sensitivity analyses.

Time	Phenotype	Analysis	MDA, n/N (%)	Between-phenotype p-value
6 months	Peripheral PsA	Observed cases	68/81 (84.0%)	0.056
6 months	Axial PsA	Observed cases	46/65 (70.8%)	
6 months	Peripheral PsA	Non-responder imputation	68/84 (81.0%)	0.146
6 months	Axial PsA	Non-responder imputation	46/65 (70.8%)	
12 months	Peripheral PsA	Observed cases	64/66 (97.0%)	0.242
12 months	Axial PsA	Observed cases	47/51 (92.2%)	
12 months	Peripheral PsA	Non-responder imputation	64/84 (76.2%)	0.590
12 months	Axial PsA	Non-responder imputation	47/65 (72.3%)	

For non-responder imputation, missing MDA assessments and discontinuation before the relevant time point were classified as non-response. P-values are chi-square tests.

Supplementary Table S9. Available-case generalized estimating equation sensitivity analyses.

Outcome	Time vs. baseline	Observations / patients	Estimated mean change (95% CI)	p-value
BASDAI	6 months	132 / 69	−2.00 (−2.71 to −1.29)	<0.001
BASDAI	12 months	132 / 69	−2.67 (−3.39 to −1.96)	<0.001
ASDAS-CRP	6 months	103 / 50	−1.05 (−1.45 to −0.66)	<0.001
ASDAS-CRP	12 months	103 / 50	−1.11 (−1.51 to −0.70)	<0.001
DAPSA	6 months	254 / 136	−13.23 (−15.27 to −11.19)	<0.001
DAPSA	12 months	254 / 136	−17.90 (−20.22 to −15.59)	<0.001

Gaussian generalized estimating equations with identity link, exchangeable working correlation structure, robust standard errors, and time as a categorical variable. All available measurements were included. These analyses do not correct for potentially informative missingness.

Supplementary Figure S1. Percentage distribution of ASDAS disease activity states at baseline, 6 months, and 12 months.

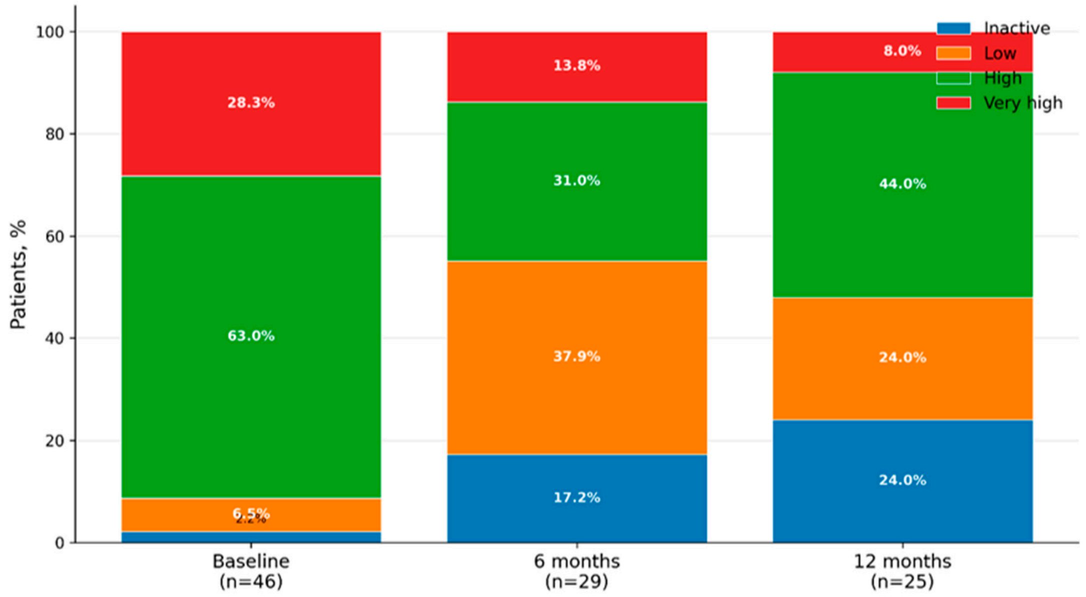

Bars represent proportions of patients with available data at each time point. Numbers within bars indicate percentages and absolute counts.

Supplementary Figure S2. Changes in ASDAS-CRP score from baseline to 6 and 12 months.

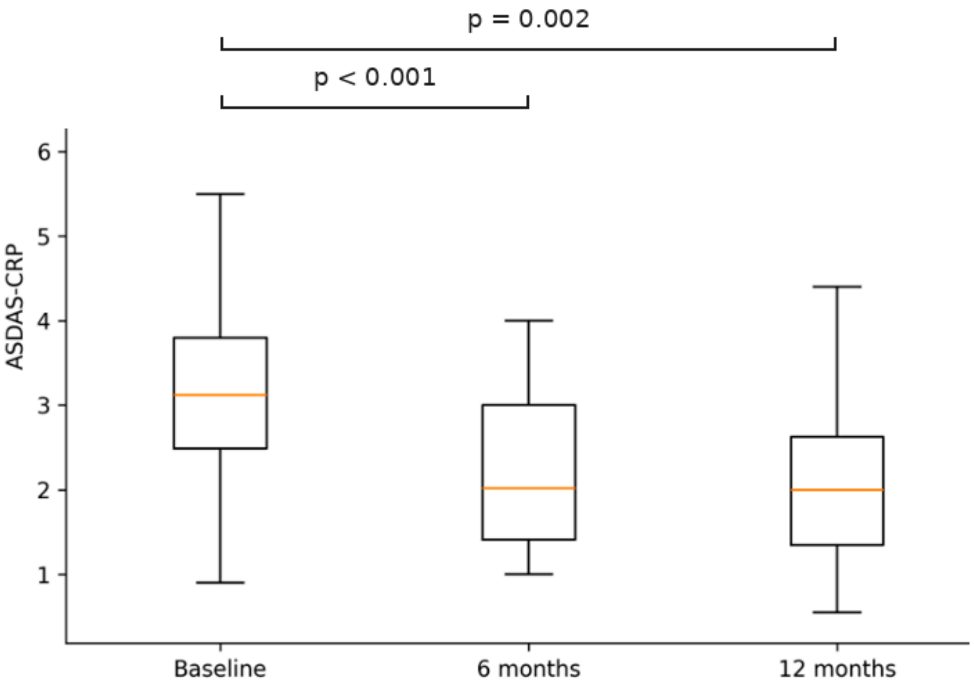

Boxplots showing the distribution of ASDAS-CRP values at baseline, 6 months, and 12 months in patients with available paired data. Boxes represent the interquartile range, horizontal lines indicate the median, and whiskers represent the range. Comparisons were performed using the Wilcoxon signed-rank test. ASDAS-CRP decreased significantly from baseline to 6 months ($p < 0.001$) and from baseline to 12 months ($p = 0.002$).
